# Relationship between Bone Mineral Density and Selected Parameters of Calcium-Phosphate Economy with Dietary Management and Metabolic Control in Polish Pediatric Patients with Classical Homocystinuria—A Preliminary Study

**DOI:** 10.3390/nu15092112

**Published:** 2023-04-27

**Authors:** Małgorzata Batycka, Ewa Lange, Ewa Ehmke vel Emczyńska-Seliga, Maciej Jaworski, Maria Kobylińska, Natalia Lech, Emilia Samborowska, Patryk Lipiński, Barbara Perkowska, Paulina Pokora, Dariusz Rokicki

**Affiliations:** 1 Department of Pediatrics, Nutrition and Metabolic Diseases, Children’s Memorial Health Institute, 04-730 Warsaw, Poland; m.batycka@ipczd.pl (M.B.);; 2 Department of Dietetics, Institute of Human Nutrition Sciences, Warsaw University of Life Sciences, 02-787 Warsaw, Poland; 3 Laboratory of Densitometry, Department of Biochemistry, Radioimmunology and Experimental Medicine, Children’s Memorial Health Institute, 04-730 Warsaw, Poland; 4 Laboratory of Fundamental Research, Department of Biochemistry, Radioimmunology and Experimental Medicine, Children’s Memorial Health Institute, 04-730 Warsaw, Poland; 5 Laboratory of Metabolism Defects, Department of Biochemistry, Radioimmunology and Experimental Medicine, Children’s Memorial Health Institute, 04-730 Warsaw, Poland

**Keywords:** homocystinuria, densitometry, bone mineral density, vitamin D3, calcium, low-protein diet, homocysteine, methionine

## Abstract

Background: Classical homocystinuria (HCU) is an inborn defect of methionine metabolism caused by a deficiency of the enzyme cystathionine β-synthase (CBS). The main symptoms of classical homocystinuria are lens subluxation, bone lesions, vascular disease and developmental delay/intellectual disability. The treatment method for HCU is a methionine-poor diet supplemented with amino acid preparations. The aim of the study was to examine the relationship of dietary factors, metabolic compensation and selected skeletal parameters in patients with HCU. Methods: Bone mineral density measurements (DXA) were performed in pediatric patients with HCU, and blood levels of selected amino acids, minerals and vitamins, as well as dietary nutritional value, were analyzed. Results: A total of 11 patients with HCU whose median age was 9.3 years were enrolled in the study. The median DXA total body less head of HCU patients was −0.4 z-score, and the lumbar spine was −1.4 z-score. Despite supplementation, calcium intake was below the age norm. Average vitamin D3 intake was in line with recommendations, but 36% of patients had reduced blood levels. Bone mineral density depended on blood levels of 25-hydroxyvitamin D, homocysteine and methionine, as well as on BMI, age and intake of natural protein (R^2^ = 98.5%, *p* = 0.015; R^2^ = 86.7%, *p* = 0.0049) and protein from an amino acid preparation (r = 0.69, *p* = 0.026). Conclusion: The results of the study indicate the need for regular densitometry in patients with HCU and also the use of additional calcium and vitamin D3 supplementation. It is also necessary to perform a comprehensive analysis of the diet and metabolic controls.

## 1. Introduction

Classical homocystinuria (HCU, # 236200) belongs to a group of rare diseases:inborn errors of metabolism classified as aminoacidopathies. It is an autosomal recessive disorder affecting the catabolism of the sulfur amino acid methionine caused by cystathionine β-synthase (CBS) enzyme deficiency associated with biallelic pathogenic variants in the *CBS* gene. Consequently, elevated levels of methionine and homocysteine in the blood/plasma and urine as well as accumulation of homocysteine in various tissues are observed [1,2]. The incidence of homocystinuria worldwide is approximately 1:200,000 births [3].

Since December 2013, all newborns in Poland are screened for classical homocystinuria (newborn screening program). However, false negative results could be obtained due to assessing the methionine concentration only as a first-tier test from a dried blood spot.

In those cases, the disease is usually diagnosed later as a result of selective screening tests, including plasma/serum aminoacidogram, with elevation in both methionine and total homocysteine levels [1,4].

Clinical symptoms of HCU include short-sightedness (an early sign of the disease), dislocation or retraction of the lens of the eye, Marfan-like appearance, epilepsy, thromboembolic events, osteoporosis, intellectual disability and psychiatric and behavioral disorders [1,3].

The first-line treatment method for HCU is a methionine-restricted diet supplemented with protein replacers (without methionine) [5]. Protein exclusion from natural products, including meat, fish, eggs, milk and dairy products, grain products, seeds, nuts and legumes, is obligatory [5,6]. Low-protein substitutes for classic foods (flour, bread, pasta, etc.), vegetables, fruits, fats and sugar can be consumed. In view of the maximum limitation of protein from classical foods, it is necessary to introduce a protein-substitute (amino acid) preparation that does not contain methionine. The preparation allows the diet to be supplemented with all other amino acids necessary for the proper functioning of the body, as well as vitamins and minerals. The amount of allowed natural protein in the patient’s diet and that from the preparation is determined by the dietitian based on the reference ranges of allowed protein intake for HCU and blood levels of methionine and homocysteine, the patient’s age and the patient’s weight (Table 1) [7].

The purpose of reducing the supply of natural protein in the diet is to lower the serum concentration of methionine and homocysteine, thereby reducing their negative effects on the body. However, some deficiencies of vitamins and minerals such as B vitamins, iron, calcium, copper, zinc and essential fatty acids may occur [10,11]. Due to calcium and vitamin D deficiencies, skeletal problems, including osteopenia and osteoporosis, can be observed [3,12,13]. When the diet, along with the supply of protein replacers enriched with vitamins and minerals, is well planned and followed by the patient, they can achieve proper metabolic control. It also affects the normal nutritional status and physical development of children and improves the quality of patients’ lives [11,14,15].

Supplementation with vitamin B6, B12 and folic acid, which are involved in methionine remethylation, is also used in HCU patients [1]. Some patients receive an anhydrous betaine (Cystadane^®^), which participates in the remethylation of homocysteine to methionine and helps to maintain normal homocysteine values [2].

The aim of this study was to examine the relationship between bone mineral density and selected parameters of calcium-phosphate economy with dietary management and metabolic control in Polish children with classical homocystinuria.

## 2. Materials and Methods

### 2.1. Study Design

A retrospective observational single-center (Children’s Memorial Health Institute, Warsaw, Poland) study was performed in 2021. A total number of 11 patients with classical homocystinuria, remaining under dietary and metabolic care, were enrolled. Dietary recommendations were made on the basis of the patient’s age, weight, laboratory results and dietary standards for HCU patients regarding the amount of natural protein and protein derived from an amino acid preparation, e.g., HCU Anamix, HCU Lophlex, HCU Cooler and HCU Express [5,8,9].

### 2.2. Assessments

Dietary assessment was performed on the basis of a 24 h dietary intake history, including amino acid preparations and supplementation used, immediately prior to examination of blood levels of selected vitamins, minerals and amino acids and whole skeletal and lumbar spine densitometry. At the same time, height and weight were measured in all patients using a stadiometer (Holtain, Harpenden Portable Stadiometer) and a scale (Radwag C 315.O). The measurement of body height was conducted without shoes, headgear and pinned-up hair for girls. The patient stood upright with their arms along the body, their back to the stadiometer, and their head, shoulders, buttocks and heels touched the device. During the measurement of body weight, the patient stood in underwear, upright in the middle of the scale, looked ahead and distributed their body weight on both legs [16]. Weight and height were measured by metabolic dietitians on calibrated digital scales, to the nearest 0.1 cm and 0.1 kg. Body mass index (BMI) values calculated on this basis were then related to the corresponding OLA/OLAF centile grids (for Polish children aged 3 to 18 years) [17].

#### 2.2.1. Dietary Assessment

The program Diet 6.0 [18] was used to analyze the menus. The energy value and intake of selected nutrients, i.e., natural protein (derived from conventional food), protein equivalent (derived from an amino acid preparation), vitamin D3, calcium and phosphorus, were related to individual dietary recommendations and dietary standards. Vitamin D3 intake was compared to standards at the adequate intake (AI) level, and calcium and phosphorus intake was compared to standards at the recommended intake level (RDA) [8,9].

#### 2.2.2. Biochemical Measures

The second part of the study was the analysis of the results for albumin, selected vitamins, minerals and amino acids in the patients’ blood. Serum calcium and albumin concentrations were determined by colorimetric method, phosphorus by photometric method, vitamin D (25OHD) by chemiluminescence method and amino acid concentrations by ion exchange chromatography (EIC). The values of the aforementioned parameters were related to laboratory standards according to the child’s age [19]. Blood serum collections were performed fasting, and analyses were made in the Laboratory of Basic Research (albumin, calcium, phosphorus) and in the Department of Biochemistry, Radioimmunology and Experimental Medicine (methionine, homocysteine, vitamin D3) at the Children’s Memorial Health Institute.

#### 2.2.3. Assessment of Bone Mineral Density

Bone mineral density measurements were conducted with dual-energy X-ray absorptiometry method (DXA). Prodigy Advance densitometer (GE Healthcare, Madison, WI, USA), with software v. 14.0 was used. Scan modes were chosen automatically by the software, based on body dimensions. Total body less head (TBLH) and lumbar spine (L2–L4) measurements were conducted according to the International Society for Clinical Densitometry recommendation [20]. Z-scores were calculated by the apparatus software based on combined NHANES/Lunar reference data. For the calibration of the densitometer, a daily quality control procedure as well as anthropomorphic spine phantom (Hologis, Waltham, MA, USA) scans were performed.

### 2.3. Ethical Permission

The study was conducted in full conformance with the principles of the “Declaration of Helsinki” (52nd WMA General Assembly, Edinburgh, Scotland, 3–7 October 2000) and Good Clinical Practice guidelines.

The study was retrospective, and data obtained from patients were anonymized. The study was approved by the Children’s Memorial Health Institute Bioethical Committee, Warsaw, Poland (36/KBE/2020).

Written consent was obtained for all subjects from at least one caregiver with parental responsibility, and written assent was obtained from the subject if appropriate for their age and level of understanding.

### 2.4. Statistical Analysis

Descriptive statistics were used to examine the characteristics of patients, the nutritional value of their diet, the use of protein substitutes, the concentration of albumin, ferritin, selected vitamins, minerals and amino acids in the blood and bone mineral density parameters. Data are presented as mean and standard deviation, median, minimum and maximum values, with a 95% confidence interval.

Univariate and multivariate regression analyses based on Spearman’s rank correlation test were used to determine the relationship between the studied characteristics. To describe the fitting of the model, R-squared statistics and standard estimation error were used.

The significance level of the analyses performed was taken as *p* ≤ 0.05. Analysis was conducted using Statgraphics Centurion 18.1.12 (Statgraphics Technologies, Inc., The Plains, VA, USA).

## 3. Results

### 3.1. Characteristics of the Group

**Figure 1:**
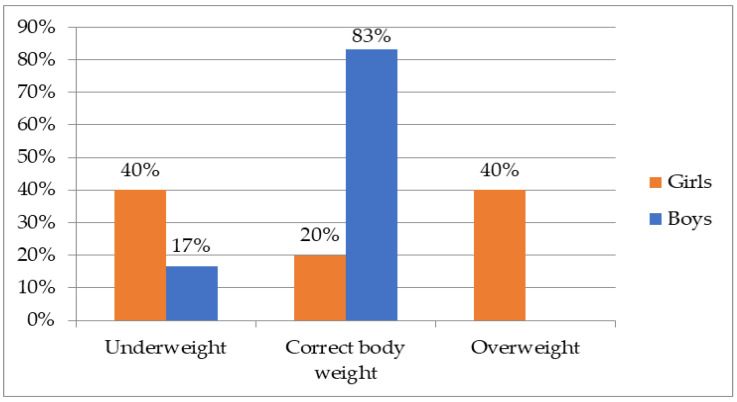
Distribution of BMI values in girls and boys with HCU (girls *n* = 5; boys *n* = 6).

The study included 11 patients (5 girls, 6 boys) with HCU aged 2.5–16 years. In only two patients, the diagnosis was obtained as a result of the neonatal screening program. Less than half of the patients (*n* = 5) were taking anhydrous betaine (Cystadane^®^). In total, 83% of HCU boys were of normal weight, while 17% were underweight. Meanwhile, 40% of HCU girls were underweight, the same percentage of girls were overweight, and only 20% were of normal weight (Figure 1).

### 3.2. Selected Parameters of Bone Mineral Density

**Figure 2:**
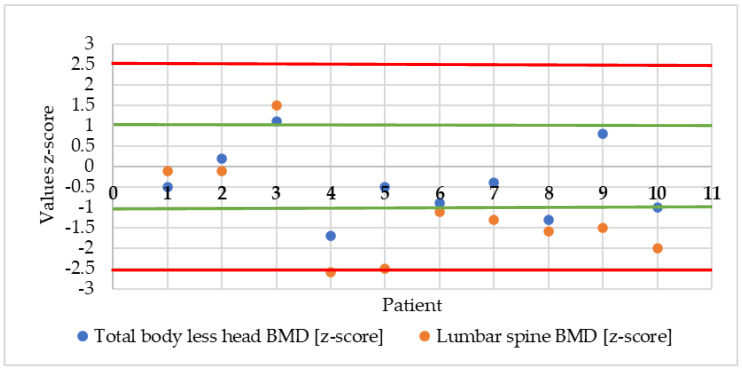
Results for bone mineral density without head and section L2–L4 [z-score]. Narrow age norm z-score from 1 to −1, upper range of broad age norm from 1 to 2.5 z-score and lower range of broad age norm from −1 to −2.5 z-score.

Ten patients with HCU were qualified for bone densitometry analysis; one patient did not meet the age criteria, as he was younger than five years old. The median z-score value of the total body (total body less head scan, TBLH) bone mineral density (BMD) was −0.5 (range −1.7 to 1.1). The median z-score value of the lumbar spine BMD was −1.4 (range −2.6 to 1.5), corresponding with the broad age range (Table 2). TBLH BMD results of six patients were within the narrow age range, one patient was within the lower limit of the narrow age range, two patients were within the lower limit of the broad age range, and one patient was within the upper limit of the broad age range. In only one girl, the bone and lumbar BMD were within the upper limit of the broad age range. The mineral density of the lumbar spine fell below the limit of the broad age range only in two patients (Figure 2).

### 3.3. Relationships between Dietary Factors, Blood Concentrations of Minerals, Vitamins and Amino Acids, as Well as Age and BMI, and Mineral Density of the Whole Skeleton Excluding Head and Lumbar Region

Multivariate regression analysis showed that in children and adolescents with HCU participating in the study, the parameters of TBLH BMD (expressed as z-score values) were dependent on natural protein, magnesium and methionine intake (R^2^ = 59.3%, *p* = 0.039). TBLH BMD was also associated with age, BMI and 25-hydroxyvitamin D levels (R^2^ = 86.7%, *p* = 0.0049). In contrast, lumbar spine mineral density indices (z-score) were dependent on 25-hydroxyvitamin D, calcium, homocysteine and methionine (R^2^ = 98.5%, *p* = 0.015). Furthermore, lumbar spine mineral density depended on protein intake from amino acid preparations (R^2^ = 48.2%, r = 0.69, *p* = 0.026) (Table 3).

In contrast, the energy value of diets was directly proportional to natural protein intake (r = +0.79, *p* = 0.004). Similarly, plasma methionine concentration was directly proportional to BMI values. A directly proportional correlation was observed for 25-hydroxyvitamin D concentration and intake with diet and supplements (R^2^ = 39.6%, r = +0.63, *p* = 0.038) (Table 4).

**Figure 3:**
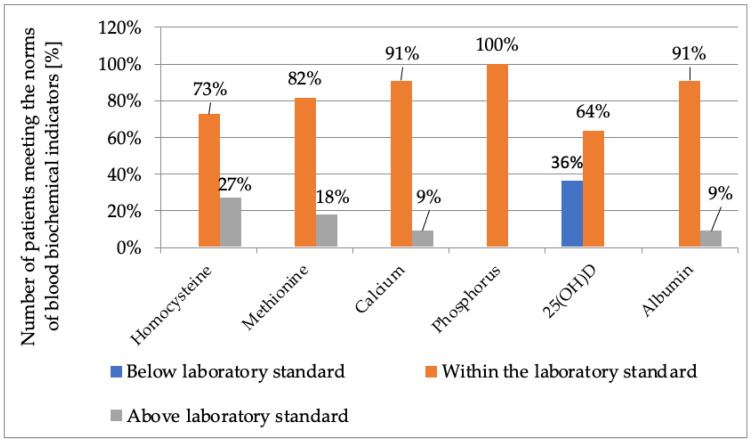
Realization of norms of blood biochemical indices in children and adolescents with HCU (n = 11).

The median value of serum albumin concentration was 44.6 g/L (range 42–48 g/L), with none of the children having a value lower than the recommended norms (Table 2, Figure 2). The median serum homocysteine level in the study group was 69.4 µmol/L (range 23.88–175.37 µmol/L), with 73% of patients having levels within the normal range and 27% having levels above normal, mostly girls. The median serum methionine level was 62.4 µmol/L (range 33.5–316.5 µmol/L), but only 2 patients had levels of this amino acid above normal. Higher levels of both homocysteine and methionine were observed in only one girl. The median serum calcium concentration was 2.4 mmol/L (range 2.31–2.63 mmol/L); only 1 boy had a calcium concentration slightly higher than the norm, and the remaining children had normal blood calcium concentrations. Similarly, all patients with HCU had normal blood phosphorus concentrations. The median 25-hydroxyvitamin D concentration was 32.3 ng/mL (range 23.9–48.1 ng/mL), with 64% of children having concentrations within the range considered optimal for this age group (30–50 ng/mL) and 36% suboptimal (20–30 ng/mL). Suboptimal 25-hydroxyvitamin D concentrations were more often observed in boys (Table 5, Figure 3).

### 3.4. Energy Value and Intake of Selected Nutrients

**Figure 4:**
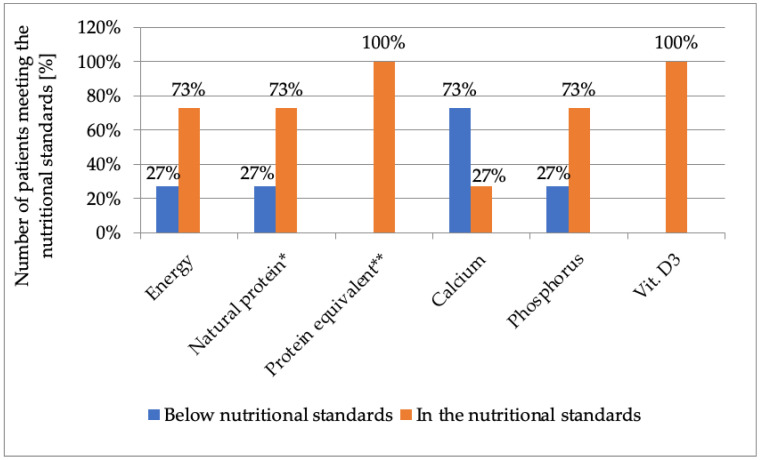
Proportion of children and adolescents with HCU meeting nutrition standards and dietary recommendations for energy, protein, calcium, phosphorus and vitamin D_3_ (n = 11). * dietary protein; ** protein from amino acid preparation.

The median dietary energy intake was 1527 kcal/d (range 875–2447 kcal/d), with 2/3 of HCU patients meeting the recommendations for dietary energy supply, but the dietary energy value of 27% of patients, mainly girls, was lower than dietary recommendations. Similarly, recommendations for natural (dietary) protein intake were met by 73% of patients (median—8.3 g/d; range 6.2–35.09 g/d), and only 2 girls and 1 boy consumed less than recommended protein intake from natural sources, 2 of whom had abnormal blood homocysteine levels. In contrast, all patients realized recommended protein intake derived from an amino acid preparation (20 g/d; range 10–45 g/d). In turn, despite the supplementation used, 73% of patients did not meet the standard for dietary intake of calcium (RDA 1000 mg/d) (631 mg/d; range 473–1496 mg/d). Vitamin D3 intake was in line with Polish nutritional recommendations (AI 15 µg/d vs. 47.26 µg/d; 17.41–97.76 µg/d). It was observed that 2/3 of children and adolescents with HCU met the recommendations for dietary intake of phosphorus (701 mg/d; 397.7–1013 mg/d) (Table 6, Figure 4).

## 4. Discussion

The analysis of collected data shows that BMD significantly lower than 0 (the expected mean Z-score in the population) can be observed in Polish children and adolescents with classical homocystinuria. In the study of Lim and Lee (2012) on five Korean pediatric patients with HCU, the mean lumbar mineral density z-score was reduced but was within the narrow age norm [13]. Similarly, in our study, the whole BMD without head (TBLH) was within the lower ranges of the narrow age norm. In the study of Weber et al. among 19 children and adults with HCU, the result fell within a broad normal range but was similar to our study [12]. However, most of the lumbar spine mineral density results in young, Polish patients with HCU were in the lower range of the broad normal range. In patients with HCU, most of the previous studies that evaluated BMD observed normal levels of methionine and homocysteine and thus good metabolic control [12,21,22]. In our study, four-fifths of patients had normal methionine levels, and three-fourths of children had normal homocysteine levels. There was a relationship found between the levels of methionine, homocysteine in the blood and the mineral density of the lumbar spine (L2–L4).

There is a known relationship between higher homocysteine levels and an increased risk of bone complications, including osteopenia and osteoporosis in both children and adults with HCU [12,23,24,25]. Such a relationship is also observed in healthy individuals [12]. The probable effect of homocysteine on the risk of osteopenia or osteoporosis is associated with a decrease in collagen cross-links in bone [12]. This causes changes in the bone matrix, which increases bone fragility and fracture risk [24]. Bone mineralization is also negatively affected by vitamin D3 and calcium deficiency [26], as well as insufficient protein intake and too little physical activity [27,28,29], although there were no fractures observed in group patients from the study.

A negative correlation between blood methionine and homocysteine concentrations and BMI values was reported in the literature [30,31]. In our study, we observed a relationship between methionine levels and BMI, as well as between BMI, age, blood 25-hydroxyvitamin D levels and BMD expressed as TBLH. More than half of the patients had a normal BMI, but weight deficiency was observed in about 30% of patients. The patient with the highest body weight had the best densitometry results.

Vitamin D3 plays an important role in bone mineralization. Among other things, it is responsible for the intestinal absorption of calcium and phosphorus [28]. All the HCU patients had a normal supply of vitamin D3 provided by the diet, amino acid preparation and supplementation. However, suboptimal blood 25-hydroxyvitamin D levels were observed in about two-fifths of the patients [26]. In a study by Weber et al., the mean blood 25-hydroxyvitamin D levels was also found to be suboptimal (28.6 ng/mL) [12]. This may have been related to the non-compliance with supplementation recommendations. The same results were obtained by Modan-Moses et al. in a study of adult patients with phenylketonuria (PKU); PKU is treated by low-phenylalanine (low-protein) diet [32]. In contrast, in a study by Kose and Arslan (2019), 60% of patients had blood 25-hydroxyvitamin D levels below 20 ng/mL [33]. In our study, we found a relationship between vitamin D3 intake and blood 25-hydroxyvitamin D levels, as well as between blood 25-hydroxyvitamin D levels and the mineral density of the entire TBLH and lumbar region. Geiger et al. found no significant correlation between vitamin D3 intake and BMD in PKU patients [29]. Most vitamin D3 is synthesized dermally by sunlight. In Poland, the possibility of such synthesis occurs mainly in spring and summer. This is one of the reasons for vitamin D3 deficiency in about 90% of adults, adolescents and children in this region [4]. This may also be one of the reasons for blood 25-hydroxyvitamin D levels deficiency in Polish HCU patients.

Calcium is the main component of bone tissue and is essential for proper mineralization of the skeleton. When there is an insufficient supply of calcium from the diet, the body begins to release calcium from the bones, which results in impaired bone mass gain and consequently increases the risk of fractures [28]. Patients with HCU in our study, despite calcium supplementation, had a lower calcium supply from the diet with respect to Polish dietary recommendations for this age group [9]. A study by Geiger et al. among patients with (PKU) showed a relationship between calcium intake and lumbar spine mineral density [29]. In our study, no such relationship was observed.

A low-protein diet, despite the use of amino acid supplements that are fortified with vitamins and minerals, can be a deficient diet [27,34]. These relationships indicate the need to select appropriate doses of vitamin D3 and calcium supplementation and to monitor their intake with the diet. These relationships may support the thesis that good metabolic control, adherence to a methionine-restricted diet and the use of calcium and vitamin D3 supplementation will have a positive effect on bone mineral density in patients with HCU [12,13].

In our study, 73% of patients were found to meet the recommendations for natural protein intake with the diet, while the rest consumed too little. The diet of the vast majority of children was of normal energy content. A multivariate correlation analysis showed that natural protein intake was dependent on the energy content of the diet. A possible explanation for this relationship may be observed due to inclusion of food such as French fries and chips, which provide protein in addition to a large number of calories. Our study showed a directly proportional relationship between protein intake from an amino acid preparation and lumbar spine mineral density. The same relationship was observed in a study by Geiger et al. conducted among patients with PKU [29]. This may indicate that consumption of amino acid preparations, which are fortified with vitamins and minerals and supplement the diet with protein, play a major role in the normal development of bone mass [29]. A study by Stroup et al. among 15 adult and adolescent patients with PKU showed the opposite—a negative relationship between the intake of amino acid preparations and bone mineral density in males [27]. The authors explained this correlation by higher urinary calcium excretion. However, the study group was too small to unequivocally confirm this thesis.

The present study was limited by several factors. One was the small study group, which is related to the rarity of classical homocystinuria in Poland. Other limiting factors were the lack of follow-up over a longer period of time and the lack of a reference group of appropriately selected children/young adults without HCU. Despite the limiting factors, this is the first such study in Poland in a group of patients with HCU, and this indicates the need for monitoring and further research.

## 5. Conclusions

Due to the restrictive diet of patients with classical homocystinuria and the consequent possibility of nutritional deficiencies, there is an increased risk of osteopenia and osteoporosis. In Polish pediatric HCU patients, bone mineralization was unsatisfactory. The diet with supplementation of most patients with respect to standards provided adequate levels of energy, protein and vitamin D3 but not enough calcium. The results for albumin, calcium, phosphorus, homocysteine and methionine blood levels were normal in the vast majority of cases, but lower than optimal blood 25-hydroxyvitamin D levels were observed. Patients with HCU need regular densitometric measurements, as well as dietary analysis for calcium and vitamin D3, and protein from both natural sources and methionine-free amino acid preparations. The above results indicate the need for additional supplementation with the aforementioned components in doses adapted to measurements of bone mineral density and their intake from the diet.

## Figures and Tables

**Table 1:** Comparison of allowed protein intake in patients with homocystinuria and in healthy population according to Polish recommendations [8,9].

Age (Years)	Polish Nutrition Standards (PZH 2020) ^1^	Individuals with Classical Homocystinuria (HCU) ^2^
	Total Protein Intake	Total Protein Intake ^2^
0–0.5	1.52 g/kg bw ^3^	2.1–2.3 g/kg bw
0.5–1	1.60 g/kg bw	2.0 g/kg bw
1–3	1.17 g/kg bw	22 g/d
4	1.1 g/kg bw	
5		32 g/d
6		
7		
8		40 g/d
9		
10		
11		
15		55–60 g/d
16	0.95 g/kg bw	50–60 g/d
18		
19	0.90 g/kg bw	

^1^ Nutrition standards at the level of adequate and recommended intake for the Polish population; ^2^ natural protein and protein from amino acid preparations; ^3^ body weight.

**Table 2:** Skeletal analysis of children and adolescents with HCU (DXA of the whole skeleton and lumbar spine, Z-score).

	n	Mean	SD	Median	Range		Confidence Intervals (CI: 5; 95)
Whole bone mineral density without head [z-score]	10	−0.42	0.9	−0.5	−1.7	1.1	−1.02; 0.18
Mineral density of the spine in the L2–L4 section [z-score]	10	−1.13	−1.3	−1.4	−2.6	1.5	−2.03; 0.23

**Table 3:** Analysis of the relationship of bone mineral density to selected dietary factors, blood concentrations of minerals, vitamins and amino acids, and age and BMI values in children and adolescents with HCU.

	Mineral Density of Whole Bone without Head [z-Score]				Mineral Density of the L2–L4 Section [z-Score]			
	F-Ratio	*p*-Value	R^2^	Standard Error of Estimation	F-Ratio	*p*-Value	R^2^	Standard Error of Estimation
Natural protein intake [g/d]	5.37 ^1^	0.0390	59.3%	0.57				
Magnesium intake [mg/d]								
Methionine intake [mg/d]								
Age [years]	15.68 ^1^	0.0049	86.7%	0.325				
BMI [kg/m^2^]								
25-hydroxyvitamin D concentration in blood [ng/mL]								
25-hydroxy vitamin D concentration in blood [ng/mL]					101.37 ^1^	0.0015	98.5%	0.152
Calcium concentration in blood [mmol/L]								
Homocysteine concentration in blood [µmol/L]								
Methionine concentration in blood [µmol/L]								
Protein intake from amino acid preparation [g/d]					7.44 ^2^	0.0260	48.2%	0.960

^1^ multivariate correlation; ^2^ univariate correlation.

**Table 4:** Analysis of the relationship of selected dietary factors, blood concentrations of minerals and amino acids, and BMI in children and adolescents with homocystinuria ^1^.

	25-Hydroxyvitamin D Concentration in Blood [ng/mL]				Energy Content of Diet [kcal/d]				BMI [kg/m^2^]			
	F-Ratio	*p*-Value	R^2^	Standard Error of Estimation	F-Ratio	*p*-Value	R^2^	Standard Error of Estimation	F-Ratio	*p*-Value	R^2^	Standard Error of Estimation
Vitamin D intake ^2^ [µg/d]	5.90	0.038	39.6%	6.54								
Natural protein intake [g/d]					14.8	0.004	72.2%	322.89				
Methionine concentration in blood [µmol/L]									18.1	0.001	81.9%	1.97

^1^ univariate regression analysis; ^2^ intake with diet and with dietary supplements.

**Table 5:** Serum concentrations of selected vitamins, minerals and amino acids in children and adolescents with HCU.

Parameter Examined	n	Mean	SD	Median	Range		Confidence Intervals (CI: 5; 95)
Homocysteine (µmol/L)	11	69.9	46.2	69.4	23.88	175.37	36.88; 102.92
Methionine (µmol/L)	11	83	79.5	62.4	33.5	316.5	26.15; 139.85
Calcium (mmol/L)	11	2.4	0.1	2.4	2.31	2.63	2.33; 2.47
Phosphorus (mmol/L)	11	1.5	0.1	1.5	1.31	1.67	1.41; 1.59
25-hydroxyvitamin D (ng/mL)	11	34.0	8.0	32.3	23.9	48.1	28.29; 39.71
Albumin (g/L)	11	44.6	1.8	44.6	42	48	43.2; 46

**Table 6:** Dietary intake of energy, protein, calcium, phosphorus and vitamin D_3_ by children and adolescents with HCU.

Dietary Intake	n	Mean	SD	Median	Range		Confidence Intervals (CI: 5; 95)
Energy (kcal/d)	11	1526.6	498.26	1448	875	2447	1191.9; 1861.3
Protein from diet (g/d)	11	12.68	9.41	8.3	6.2	35.09	6.38; 18.98
Protein from amino acid preparations (g/d)	11	24.34	9.08	20.0	10.0	45.0	18.24; 30.44
Calcium intake from diet + supplementation (mg/d)	11	759.1	333.2	631.0	473.0	1496.0	535.3; 928.9
Phosphorus intake from diet (mg/d)	11	708.5	186.1	701.0	397.7	1013.0	583.4; 833.6
Vitamin D_3_ from diet + supplementation (µg/d)	11	46.63	24.22	47.26	17.41	97.76	30.33; 62.93

## Data Availability

Data available from the authors: ewa_lange@sggw.edu.pl and m.batycka@ipczd.pl.
